# Training community healthcare workers on the use of information and communication technologies: a randomised controlled trial of traditional versus blended learning in Malawi, Africa

**DOI:** 10.1186/s12909-018-1175-5

**Published:** 2018-04-02

**Authors:** Nikolaos Mastellos, Tammy Tran, Kanika Dharmayat, Elizabeth Cecil, Hsin-Yi Lee, Cybele C. Peng Wong, Winnie Mkandawire, Emmanuel Ngalande, Joseph Tsung-Shu Wu, Victoria Hardy, Baxter Griphin Chirambo, John Martin O’Donoghue

**Affiliations:** 1 Global eHealth Unit, Department of Primary Care and Public Health, School of Public Health, Imperial College London, Reynolds Building, London, W6 8RP UK; 2 Dr Foster Unit, Department of Primary Care and Public Health, School of Public Health, Imperial College London, London, UK; 3 Luke International, Mzuzu, Malawi; 4 Department of Information and Communication Technology, Mzuzu University, Mzuzu, Malawi; 5 Department of Family Medicine, University of Washington, Seattle, WA USA; 6 Faculty of Health Sciences, Mzuzu University, Mzuzu, Malawi

**Keywords:** Community healthcare workers, Developing countries, Blended learning, Traditional learning, mLearning, eHealth, Information and communication technologies

## Abstract

**Background:**

Despite the increasing uptake of information and communication technologies (ICT) within healthcare services across developing countries, community healthcare workers (CHWs) have limited knowledge to fully utilise computerised clinical systems and mobile apps. The ‘Introduction to Information and Communication Technology and eHealth’ course was developed with the aim to provide CHWs in Malawi, Africa, with basic knowledge and computer skills to use digital solutions in healthcare delivery. The course was delivered using a traditional and a blended learning approach.

**Methods:**

Two questionnaires were developed and tested for face validity and reliability in a pilot course with 20 CHWs. Those were designed to measure CHWs’ knowledge of and attitudes towards the use of ICT, before and after each course, as well as their satisfaction with each learning approach. Following validation, a randomised controlled trial was conducted to assess the effectiveness of the two learning approaches. A total of 40 CHWs were recruited, stratified by position, gender and computer experience, and allocated to the traditional or blended learning group using block randomisation. Participants completed the baseline and follow-up questionnaires before and after each course to assess the impact of each learning approach on their knowledge, attitudes, and satisfaction. Per-item, pre-post and between-group, mean differences for each approach were calculated using paired and unpaired t-tests, respectively. Per-item, between-group, satisfaction scores were compared using unpaired t-tests.

**Results:**

Scores across all scales improved after attending the traditional and blended learning courses. Self-rated ICT knowledge was significantly improved in both groups with significant differences between groups in seven domains. However, actual ICT knowledge scores were similar across groups. There were no significant differences between groups in attitudinal gains. Satisfaction with the course was generally high in both groups. However, participants in the blended learning group found it more difficult to follow the content of the course.

**Conclusions:**

This study shows that there is no difference between blended and traditional learning in the acquisition of actual ICT knowledge among community healthcare workers in developing countries. Given the human resource constraints in remote resource-poor areas, the blended learning approach may present an advantageous alternative to traditional learning.

**Electronic supplementary material:**

The online version of this article (10.1186/s12909-018-1175-5) contains supplementary material, which is available to authorized users.

## Background

In recent years, community healthcare workers (CHWs) have re-emerged as an essential component of the health workforce needed in order to meet the health-related Millennium Development Goals (MDGs) [1]. By linking the gap between the health system and rural communities, CHWs have become the cornerstone of primary health care in many developing countries, where lack of human resources in remote areas often hinders healthcare systems from delivering effective evidence-based care to rural communities. In Malawi, 83% of the population lives in rural areas, which shifts many of the primary care responsibilities, such as family planning, immunisation and management of common childhood illnesses (e.g. malaria, pneumonia and diarrhoea), to the cadre of CHWs [2]. Their essential role in the national health system is recognized in the Malawi’s Community Health Strategy (2017–2022), which also highlights the need for strengthening the community health information system by using information and communication technologies (ICT) [3].

The utilization of ICT is considered to be one of the most important modes to improving the quality of healthcare services in both developed and developing countries [4]. Since 2006, the Malawian Ministry of Health, in collaboration with Baobab Health Trust and Luke International, two non-governmental organizations (NGOs) operating locally, have begun investing in ICT solutions by installing electronic health record (EHR) systems in health facilities throughout Malawi [5, 6]. These EHR systems aim to improve patient outcomes by assisting the management of supplies, aiding clinicians in the delivery of care, as well as providing robust patient-level data for stakeholders. In addition, mobile phone penetration among community health workers has risen remarkably. A recent assessment of mobile phone penetration rate across five districts in Malawi found that mobile phone penetration among CHWs is approaching 100%. Smartphone ownership was around 80% among decision-makers in health facilities, and 50% among CHWs, data clerks and other data handlers [7].

Digital solutions have also been implemented to strengthen supply chains and improve access to medicines by healthcare professionals. In 2008, the government of Malawi initiated the integrated Community Case Management (iCCM) strategy, which seeks to deliver care and treatment to children with common childhood illnesses, particularly in rural communities [8]. A major component of CHWs’ success is the access to medical supplies, which is often hindered by poorly functioning supply chains. To address the constraints in the supply chain, cStock, a mobile health (mHealth) tool for community-level reporting of stock was implemented. CHWs report stock on hand through short message service (SMS) to cStock, which calculates their resupply quantity and immediately informs the health centre via SMS. Health centres can then advise CHWs when stock is available for collection. This mHealth solution provided improvements to data visibility across all levels of the health system, and has resulted in reduced supply stockouts [9].

With over 5.4 million individual mobile phone users in Malawi (34% of the population), the potential for mHealth solutions is increasingly gaining support from governmental bodies, NGOs, and researchers [10]. While SMS-based mHealth interventions have become progressively popular, there has also been an increased use of smartphones as health solutions. Multiple studies conducted in Malawi utilize smartphone applications to monitor and record patients [11] as well as aid in clinical decision-making [12]. While these studies are pioneering the initiative for mHealth in developing countries, these types of interventions require government stewardship and stable infrastructure, among other things, to become sustainable [13].

As many technologies have not yet fully penetrated rural communities, CHWs may have limited exposure to devices, such as smartphones or computers. At present, CHWs and their supervisors, do not receive ICT or computer skills training as part of their professional development [14]. In order for healthcare systems to fully benefit from mHealth solutions, CHWs should have at least basic computer skills and knowledge. However, the vast majority of healthcare workers in rural settings are not computer literate [15]. By providing CHWs with introductory computer skills training, they can become equipped with the necessary knowledge and confidence to use such technologies.

As the majority of CHWs live and work in off-grid communities, they would be required to travel to urban cities to attend training courses. While attending a training or refresher course, they would be required to take a leave of absence for the duration of the course, which would come at a cost in welfare to their communities [16]. On average, in Malawi, one CHW is responsible for four to five villages, which equates to approximately 1000 community members [17]. If one CHW is away from his or her community for one to two weeks at a time, thousands of community members may be left without access to primary healthcare.

With such constrained resources, there are many challenges to be addressed before CHWs can access education and training. Therefore, eLearning may be able to overcome some of the challenges or barriers that deter CHWs from attending traditional learning courses [18]. eLearning refers to the learning process created or supported by the use of digital technologies to create, deliver, and facilitate learning [19]. As technologies become more readily available, modes of learning have also adapted to take advantage of such innovations. eLearning courses have become commonplace in many institutions due to the unique advantages it offers over traditional learning styles, such as increased accessibility to information, better content delivery, on-demand availability, self-pacing, and potentially reduced costs [20]. However, successful eLearning relies on students having the knowledge and skills to use computers or mobile devices. While CHWs would benefit from the advantages of eLearning, it would not yet be feasible for them to effectively learn how to operate computers and programmes through distance eLearning. Basic computer skills, such as using a keyboard, viewing documents, and saving files, are often taught with practical experience-based learning models [21]. Since most community health workers do not own or have access to a computer, they would be required to travel to an urban area to access a computer. Therefore, a traditional classroom environment, where content is delivered through face-to-face instruction, would very beneficial in the context of teaching computer skills in Malawi, but would require CHWs to leave their communities for an extended period of time.

Alternatively, a blended learning approach offers a solution that addresses the challenges of traditional learning and eLearning. Blended learning has been found to produce significant savings for training CHWs in Sub-Saharan Africa as a result of decreased classroom time and associated cost reductions for travel, trainers and classroom [22]. This approach incorporates a combination of traditional face-to-face learning with other types of content delivery, often using digital media, to promote a more effective learning experience [23]. Because blended learning models can be adapted to meet the needs of the learners, courses can be adapted to optimize benefits from both traditional learning and eLearning or mLearning (mobile learning) models for the learners. Due to infrastructural limitations in Malawi, CHWs (known in Malawi as Health Surveillance Assistants or HSAs) would be limited to mLearning rather than computer-based eLearning. While the traditional learning component could offer the practical experience needed for computer literacy, the mLearning component could reduce the costs of absenteeism from their community responsibilities.

The focus of this paper is on investigating the feasibility of integrating distance mLearning as part of a blended learning programme in Malawi, Africa. In doing so, a pilot course was organised with the aim to develop and validate two questionnaires measuring CHWs’ knowledge of and attitudes towards the use of ICT, as well as their satisfaction with each course (phase 1). Those were then used in a trial assessing the effectiveness of the traditional and blended learning “Introduction to ICT and eHealth” courses (phase 2). More specifically, the objectives of the work described in this paper were:

- To develop and test a questionnaire assessing HSAs’ knowledge and attitudes towards computers, tablets and smartphones (phase 1).
- To assess the effectiveness of the traditional and blended learning courses in terms of improving HSAs’ knowledge and attitudes towards computers, tablets and smartphones (phase 2).
- To assess participants’ experience and satisfaction with the courses (phase 2).

## Methods

### Phase 1

#### Questionnaire development

Two questionnaires (pre- and post-) were developed and tested during a pilot course at Mzuzu University in November 2015. The questionnaires were designed to measure HSAs’ self-rated ICT skills (thereafter referred to as self-rated ICT knowledge), actual ICT skills (thereafter referred to as ICT knowledge) and attitudes (i.e. positive or negative beliefs about the usefulness and ease of use of, as well as self-efficacy with, computers, tablets and smartphones) towards using computers, tablets and smartphones. Their development was informed by literature and expert review from a multidisciplinary group of researchers with clinical, computer science and eLearning backgrounds. The literature review included studies measuring HSAs’ knowledge, attitudes and satisfaction with similar initiatives in Sub-Saharan Africa [15, 24–26].

The pre-questionnaire was designed to collect socio-demographic data (i.e. age, gender, educational and marital status, religion, income, work location, position, experience and job satisfaction), and information about HSAs’ experience with computers, tablets and/or smartphones. Three scales were developed to measure participants’ knowledge and attitudes towards using computers, tablets and smartphones. The self-rated ICT knowledge scale is a 10-item, 5-point Likert scale measuring respondents’ perceived ICT knowledge (5 = “Strongly agree”, 1 = “Strongly disagree”). The ICT knowledge scale is a 10-item, multiple answer scale assessing HSAs’ knowledge of using computers, tablets and smartphones. The scale was designed to compare HSAs’ perceptions of their ability to use computers, tablets and smartphones (self-rated ICT knowledge scale) to their actual knowledge of using those devices. Finally, the attitudes scale is a 10-item, 5-point Likert scale measuring HSAs’ attitudes towards computers, tablets and smartphones (5 = “Strongly agree”, 1 = “Strongly disagree”). The scale includes an additional “Do not know” answer option for those participants with no previous knowledge of or experience with using computers (Additional file 1).

The post-questionnaire was developed to measure HSAs’ knowledge and attitudes after the course using all but the socio-demographic data and computer experience scales from the pre-questionnaire. The questionnaire also includes a 10-item, 5-point Likert scale (5 = “Strongly agree”, 1 = “Strongly disagree”) measuring HSAs’ satisfaction with the course (Additional file 2). Each questionnaire requires about 10–15 min to complete. A higher score indicates greater knowledge, attitudes, and satisfaction. Reversed items were converted for scoring.

#### Questionnaire validation

The face validity of the scales was tested with seven HSAs in Mzuzu, separate to those recruited to the trial. Overall, participants felt that the questions were clear and easy to answer. Most HSAs were able to complete the questionnaires without asking any questions, while two asked for clarifications during the testing. Based on their feedback, two changes were made. First, a “Do not know” option was added for all questions in the ICT knowledge and attitudes scales, as it was suggested that HSAs with no previous experience with computers, tablets or smartphones would be unable to provide an answer. Second, instead of listing all Christian denominations separately, one option for all participants of Christian faith was given with an additional “specify denomination” field. The reliability of the self-rated ICT knowledge and attitudes scales was assessed in a pilot course with the participation of 20 HSAs from urban and rural health facilities in Mzimba District. Those were selected by the DHO and were different to those participating in the trial. Researchers from Imperial College London and Luke International visited the HSAs at their workplace and asked them to fill out the baseline questionnaire. Due to the large distance of HSAs’ clinics from Mzuzu, the first round of data collection was conducted on three separate days. One week later, HSAs attended a five-day, lab-based “Introduction to ICT and eHealth” course at Mzuzu University. The aim of the pilot course was to assess the appropriateness of the content and duration of the course, as well as test the reliability of the self-rated ICT knowledge and attitudes scales. At the outset of the course, participants were asked to recomplete the baseline questionnaire. One participant could not be reached before the course and thus completed the questionnaire only once, at Mzuzu University. To evaluate the internal consistency of participants’ self-rated ICT knowledge and attitudes prior to the course, Cronbach’s alphas were calculated for the 10-item perceived knowledge and attitudes ratings reported at the baseline questionnaire. Both scales demonstrated modest to high internal consistency, with Cronbach’s alphas of .940 for the self-rated knowledge and .643 for the attitudes scale, respectively. The test-retest reliability of subscales was assessed separately on the perceived ICT knowledge and attitudes, as all but one participant completed the baseline questionnaire on two occasions prior to the start of the course. Intra-class correlation coefficients (ICCs) and 95% confidence intervals (CI) for the ICCs were calculated using a one-way random effects model. The ICC of both scales indicated modest degree of reliability (self-rated ICT knowledge: ICC = .846, 95% CI = .656–.935, *p* < .001; attitudes: ICC = .449, 95% CI = .028–.736, *p* < .05).

### Phase 2

#### Study design

A randomised controlled trial study design was used to compare the effectiveness of the traditional and blended learning courses. The traditional learning course was delivered in December 2015, and the blended learning course in August 2016. Contemporaneous delivery of the courses was not feasible due to the different length of each course and the limited number of available computers (*n* = 20) at Mzuzu University in Mzuzu, a capital city in Northern Malawi.

#### Participants and setting

The study was conducted at Mzuzu University in Mzuzu. Participants were HSAs and senior HSAs. We asked the district health officer (DHO) of Mzimba District to select 30 HSAs and 10 senior HSAs from rural clinics (total number of senior/HSAs in Mzimba District = 249). The decision to recruit more HSAs was based on the premise that HSAs are directly involved in health care provision, while senior HSAs play a supervisory role and therefore would be less likely to use tablets or smartphones to deliver care. To be eligible for participation, HSAs had to be fluent in spoken and written English, have limited or no prior experience with computers, tablets or smartphones, be able to travel to Mzuzu University to attend the lab-based sessions, be agreeable to complete the pre- and post-questionnaires, and be willing to provide informed consent.

#### Interventions

The traditional learning group (control group) attended a five-day, class-based course at Mzuzu University. The design of the course was based on Bloom’s taxonomy [27] and aimed to equip HSAs and their supervisors with adequate knowledge and skills to use ICT solutions, such as computers, tablets and smartphones, in their everyday practice. The course was also designed to advance participants’ understanding of the potential of eHealth and mHealth in healthcare provision and public health policy. The course consisted of 19 face-to-face, lab-based sessions, lasting between 1 and 2 h each, giving a total of 31 learning hours. Those were delivered through lectures, seminars and training workshops by a joint faculty from Imperial College London, Mzuzu University and Luke International using PowerPoint slides, computers, tablets and smartphones. A bilingual research assistant in English and Tumbuka (language spoken in Mzimba District) was present during all sessions to support learners with difficulties to understand certain concepts in English. Participants were provided with lunch and refreshments during the course and were compensated for their travel and accommodation costs. Course delivery and attendance were monitored using a checklist and an attendance sheet, respectively. A certificate of completion was provided to all participants who attended the course.

The blended learning group received the exact same content as the traditional learning group by the same tutors, but over three weeks. A mixed (i.e. lab and flipped classroom) rotation model was used where three sessions were delivered in a computer lab on campus via eLearning and five off-site at the HSAs’ place of homework (e.g. home) via mLearning. Participants were also required to attend seven class-based, tutor-guided seminars and one training workshop at Mzuzu University. Similar to the traditional learning course, an opening, closing and discussion session were included. The course consisted of 21 face-to-face learning hours and 10 h of independent study. The eLearning content consisted of videos with script-guided lectures created with Adobe Premiere Pro and Microsoft PowerPoint, and was delivered offline due to network connectivity limitations. Participants were provided with smartphones for the off-site sessions to enable them to go through the eLearning material. Like the traditional learning group, participants were equipped with computers and smartphones for the lab and class-based sessions, were provided with lunch and refreshments, and were compensated for their travel and accommodation expenses. They finally received similar support during the on-site classes and telephone support and reminders during the off-site sessions. Table 1 provides a summary of each course using the GREET checklist [28] and adapting it to the study context.

**Table 1:** Traditional and blended learning course summary

	Traditional learning	Blended learning
Interventions	5-day class-based “Introduction to ICT and eHealth” course	3-week blended learning “Introduction to ICT and eHealth” course
Theory	Bloom’s taxonomy	Bloom’s taxonomy, mixed rotation model
Learning objectives	To equip HSAs and Senior HSAs with knowledge and skills to use computers, tablets and smartphones in their everyday practice, as well as to advance their understanding of the potential of e/mHealth in health care provision and public health policy	To equip HSAs and Senior HSAs with knowledge and skills to use computers, tablets and smartphones in their everyday practice, as well as to advance their understanding of the potential of e/mHealth in health care provision and public health policy
Content	Introduction to computers, tablets and smartphones; Computer skills; Introduction to eHealth; Introduction to mHealth; Use of community and facility-based data in decision making	Introduction to computers, tablets and smartphones; Computer skills; Introduction to eHealth; Introduction to mHealth; Use of community and facility-based data in decision making
Materials	PowerPoint slides, computers, tablets and smartphones	PowerPoint slides, computers, tablets and smartphones
Educational strategies	Lectures, seminars, tutor-led training workshops	Seminars, eLearning workshops, off-site mLearning
Incentives	Travel, catering and accommodation costs	Travel, catering and accommodation costs
Instructors	Faculty from Imperial College London and Mzuzu University, Senior staff from Luke International	Faculty from Imperial College London and Mzuzu University, Senior staff from Luke International
Delivery	Face-to-face (1:19 instructor/learner ratio)	Face-to-face (1:20 instructor/learner ratio), e/mLearning
Environment	University lab	University lab, community
Schedule	19 sessions (opening, 6 lectures, 7 seminars, 4 tutor-led training workshops, discussion, closing), delivered over 5 days (9 am-5 pm Monday-Friday), lasting between 1 and 2 h each (total learning hours = 31), with 15-min breaks between sessions and 1 h lunch break each day	19 sessions (opening, 5 mLearning sessions, 7 seminars, 1 tutor-led and 3 eLearning workshops, discussion, closing), delivered over 3 weeks (Week 1: 9 am-5 pm Monday-Tuesday, Week 2: 2 h per day Monday-Friday, Week 3: 9 am-5 pm Tuesday), lasting between 1 and 2 h each (total learning hours = 31), with 15-min breaks between sessions and 1 h lunch break each day
Face-to-face time	31 h face-to-face time	21 h face-to-face time, 10 h independent study
Adaptations	To maximize the benefit for learners a bilingual research assistant was present at all times	To maximize the benefit for learners a bilingual research assistant was present during all face-to-face sessions (including the on-site eLearning sessions) and available via phone on the days of the mLearning sessions, and an offline mLearning module was designed due to network connectivity limitations
Modifications	None	None
Attendance	Participants were required to sign an attendance sheet at the beginning and end of each day. Of the 20 participants assigned, 19 attended the course	Participants were required to sign an attendance sheet at the beginning and end of each class-based day. For the off-site learning sessions, participants were required to respond to daily telephone reminders. All 20 participants attended the course
Planned delivery	A checklist was used to determine whether the course materials and educational strategies were delivered as planned	A checklist was used to determine whether the course materials and educational strategies were delivered as planned
Actual schedule	The course was delivered as scheduled	The course was scheduled in June 2016 but was delivered in August 2016 upon request from the DHO

#### Outcomes

The primary outcomes of the study were ICT (self-rated and actual) knowledge and attitudes. Satisfaction with the course was a secondary outcome. Data on those outcomes were collected using the pre- and post- questionnaires, which were administered before (first day) and after (final day) each course.

#### Sample size

Our sample size was limited by the number of available computers at Mzuzu University. This constrained the number of participants to 20 per arm.

#### Randomisation

After receiving the anonymised baseline data for the 40 participants, a researcher from Imperial College London stratified participants by position, gender and experience with computers, tablets or smartphones to achieve equal representation among the two groups. Block randomisation was used to allocate participants to one of the study groups. A computer random number generator was used to select random permuted blocks with a block size of four and allocate 20 participants to the blended learning group and 20 to the traditional learning group.

#### Blinding

Participants were masked to the intervention. It was not possible to mask the study personnel, but outcome assessment was blinded.

#### Statistical methods

All statistical analyses were carried out using STATA (version 12.1). Data were analysed using the principle of intention-to-treat. The unit of analysis was the mean change in per item score before and after each course across all respondents in the group. Mean differences in per item (self-rated and actual) ICT knowledge and attitudes across all respondents before and after each course were compared using paired t-tests. Between-group (blended vs traditional learning) differences were explored using unpaired t-tests. Post-course, between-group, satisfaction scores for each item were compared using unpaired t-tests. All statistical tests were two-sided with the level of significance set at α = 0.05.

## Results

### Participants’ socio-demographic characteristics

**Fig. 1:**
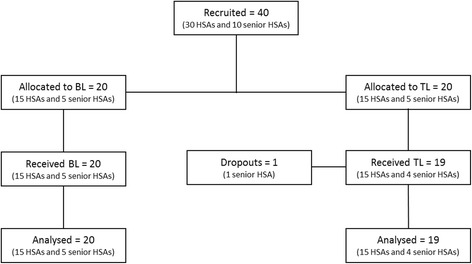
Participant flow diagram

A total of 40 HSAs were recruited in November 2015 by the DHO of Mzimba District. Of those, 20 were assigned to the traditional learning group and 20 to the blended learning group. One participant from the traditional learning group dropped out and was excluded from the analysis (Fig. 1). Participants were aged between 27 and 53 years of age, with the mean age being 40 years. Most participants (79.5%) were male due to the inequivalent gender composition of HSAs in Malawi. All participants had received some type of formal education, given that the Ministry of Health requires that all HSAs complete a Secondary Certificate Examination or Junior Certificate to be eligible for employment [17].

Nearly all participants (97.4%) received secondary education, while one participant only completed primary education. Of the 39 participants, 25 (64%) were HSAs and 14 (36%) senior HSAs who serve as supervisors. Nearly all (97.4%) participants reported to be satisfied or very satisfied with their job. Most participants did not have access to a computer nor had never been formally trained to use computers. Two participants (5%) reported to have access to a computer either at home or work or through a friend. Three participants (7.6%) had received some type of computer training in the past (Table 2).

**Table 2:** Participant characteristics

		Traditional (*N* = 19)		Blended (*N* = 20)	
		*N*	%	*N*	%
Sex	Female	4	21%	4	20%
	Male	15	79%	16	80%
Age	≤30	3	16%	0	0%
	31–39	4	21%	9	45%
	≥40	12	63%	11	50%
Education level	Primary	1	5%	0	0%
	Secondary	18	95%	20	100%
Work position	HSA	13	68%	12	60%
	Senior HSA	6	32%	8	40%
Experience in years	1 to 10	8	42%	10	50%
	11 to 20	9	47%	5	25%
	21 to 30	2	11%	5	25%
Satisfaction with job	Satisfied or very satisfied	18	95%	20	100%
	Neither satisfied nor dissatisfied	1	5%	0	0%
Access to a computer	Yes	0	0%	2	10%
	No	18	95%	18	90%
Previous computer training	Yes	1	5%	2	10%
	No	18	95%	18	90%

### Self-rated ICT knowledge

Both groups experienced significant increases in post-intervention scores compared to pre-intervention scores. Differences in self-rated knowledge scores were higher across participants in the traditional learning group compared with participants in the blended learning group, with statistically significant differences in seven domains (Table 3). However, the blended learning group had higher baseline self-rated knowledge scores compared with the traditional learning group.

**Table 3:** Self-rated ICT knowledge

	Traditional (19)			Blended (20)			Between groups
	Pre	Post	MD (95% CI)	Pre	Post	MD (95% CI)	MD (95% CI)
Question							
I know how to start and close down a computer	1.37	5.00	3.63 (3.26–3.99)	2.05	4.79	2.74 (1.97–3.50)	− 0.89 (−1.72--0.07)
I know how to distinguish between basic computer components, such as software applications, hardware and operating systems	1.11	4.95	3.84 (3.66–4.02)	1.84	4.79	2.95 (2.24–3.66)	−0.89 (−1.60--0.19)
I know how to navigate the Internet with a browser	1.11	4.72	3.61 (3.36–3.86)	2.16	3.63	1.47 (0.80–2.14)	−2.14 (−2.84--1.43)
I know how to download and save content (e.g. pdf files)	1.11	4.68	3.58 (3.33–3.82)	2.58	4.00	1.42 (0.60–2.25)	−2.16 (− 2.99--1.33)
I know how to use an email management tool (Microsoft Outlook)	1.11	2.68	1.58 (0.93–2.23)	2.11	3.89	1.79 (0.99–2.59)	0.21 (−0.78–1.20)
I know how to use a word processor (Microsoft Office Word) application	1.26	4.89	3.63 (3.26–3.99)	1.83	4.60	2.72 (2.22–3.23)	− 0.91 (−1.51--0.31)
I know how to use spreadsheets (Microsoft Office Excel)	1.11	4.67	3.56 (3.30–3.81)	1.74	4.42	2.68 (2.17–3.19)	−0.87 (−1.43--0.31)
I know how to use a visual and graphical application (Microsoft Office PowerPoint)	1.32	4.67	3.33 (2.95–3.71)	1.63	4.32	2.68 (2.20–3.17)	−0.65 (−1.25--0.05)
I know how to switch on, navigate and switch off a tablet or smartphone	2.89	5.00	2.11 (1.23–3.00)	3.80	5.00	1.20 (0.58–1.82)	−0.91 (−1.94–0.11)
I know how to navigate a mobile app	2.39	4.89	2.44 (1.68–3.21)	3.00	4.50	1.50 (0.86–2.14)	−0.94 (−1.91–0.02)

### ICT knowledge

The ICT knowledge scale was designed to assess participants’ actual knowledge on how to perform particular tasks using computers, tablets and smartphones. Participants were required to select one or more options for each question and earned one point for each completely correct response or no points for each partially correct, incorrect or blank response. Overall, post-intervention scores were higher than pre-intervention scores in both groups, apart from their understanding of an operating system, with significant pre-post differences in two domains (one per group). Statistically significant between-group differences were only found in the ability of the traditional learning group to download and save a journal article in a pdf format (Table 4).

**Table 4:** ICT knowledge

	Traditional (19)			Blended (20)			Between groups
	Pre	Post	MD (95% CI)	Pre	Post	MD (95% CI)	MD (95% CI)
Question							
The ‘start menu’ allows you to:	0	0	–	0	0.06	0.13 (−0.17–0.42)	0.13 (− 0.06–0.31)
Which of the following is an example of an operating system?	0.05	0	−0.05 (− 0.16–0.06)	0.21	0.16	− 0.05 (− 0.31–0.20)	0 (− 0.27–0.27)
Which of the following is an example of an Internet browser?	0	0	–	0	0	–	–
I can download and save a journal article as:	0	0.74	0.74 (0.51–0.95)	0.18	0.47	0.30 (−0.01–0.60)	− 0.44 (− 0.80--0.09)
I can use Microsoft Outlook or Yahoo Mail or Gmail or Hotmail to:	0	0	–	0	0.05	0.05 (−0.06–0.16)	0.05 (− 0.05–0.16)
I can use Microsoft Word to:	0	0.13	0.13 (−0.06–0.31)	0	0.07	0.07 (−0.08–0.22)	− 0.05 (− 0.29–0.18)
I can use Microsoft Excel to:	0	0.06	0.06 (− 0.06–0.31)	0	0.14	0.14 (− 0.07–0.35)	0.08 (− 0.13–0.30)
I can use Microsoft PowerPoint to:	0	0	–	0	0	–	–
can use a tablet or smartphone to:	0.05	0.10	0.05 (−0.14–0.25)	0.05	0.25	0.20 (−0.04–0.44)	0.15 (− 0.16–0.45)
I can use a health mobile app to:	0	0.11	0.11 (−0.05–0.26)	0.05	0.15	0.10 (0.04–0.24)	−0.01 (− 0.21–0.20)

### Attitudes towards computers, tablets and smartphones

Both groups experienced positive attitudinal gains after attending the course. Post-intervention attitudinal scores were predominantly high, with the blended learning group experiencing significant attitudinal gains in five domains and the traditional learning in two (Table 5). However, there were no significant differences in attitudes scores between groups.

**Table 5:** Attitudes towards computers, tablets and smartphones

	Traditional (19)			Blended (20)			Between groups
	Pre	Post	MD (95% CI)	Pre	Post	MD (95% CI)	MD (95% CI)
Question							
I believe computers, tablets and smartphones are useful in my everyday (non-work related) life	4.79	4.95	0.16 (− 0.24–0.56)	4.40	4.50	0.10 (− 0.61–0.81)	− 0.06 (− 0.86–0.74)
I believe computers and mobile apps can assist me in my work at the health centre	4.63	5.00	0.37 (0.08–0.66)	4.50	5.00	0.50 (0.26–0.74)	0.13 (− 0.23–0.49)
I believe I would be able to use a computer or mobile app to provide patient care	4.63	5.00	0.37 (0.08–0.66)	4.35	4.95	0.60 (0.32–0.88)	0.23 (− 0.16–0.62)
I believe I would be able to learn how to use a computer or mobile app	4.79	5.00	0.21 (0.05–0.47)	4.65	5.00	0.35 (0.12–0.58)	0.14 (−0.19–0.47)
I believe computers and mobile apps can support my decision making during healthcare provision	4.74	5.00	0.26 (0.01–0.53)	4.70	5.00	0.30 (0.08–0.52)	0.04 (−0.30–0.37)
I think using computers and mobile apps would increase my workload	1.74	1.74	0	1.47	1.32	−0.16 (− 0.56–0.24)	−0.16 (− 0.60–0.29)
I think the use of computers and mobile apps would improve quality of care	4.63	5.00	0.37 (−0.03–0.77)	4.55	4.95	0.40 (−0.11–0.91)	0.03 (− 0.60–0.66)
I do not have time to learn how to use computers and mobile apps	1.83	1.44	0.39 (−1.14–0.36)	1.42	1.37	−0.05 (− 0.55–0.44)	0.34 (− 0.52–1.19)
I do not have time to use computers and mobile apps	1.89	1.44	− 0.44 (− 1.25–0.36)	1.53	1.58	0.05 (− 0.44–0.55)	0.49 (− 0.40–1.40)
Overall, I believe using computers and mobile apps in patient care is a good idea	4.94	5.00	0.06 (− 0.15–0.26)	4.80	5.00	0.20 (− 0.44–0.55)	0.14 (− 0.13–0.42)

### Satisfaction with the course

Satisfaction scores were generally high in both groups. There were significant differences between groups in two domains. Participants in the blended learning group found it more difficult to follow the content of the course compared to the participants in the traditional learning group. In addition, although both groups had high satisfaction scores, participants in the traditional learning group enjoyed the course more compared with participants in the blended learning group (Table 6).

**Table 6:** Satisfaction with the course

	Traditional (19)	Blended (20)	Between groups
	Mean	Mean	MD (95% CI)
Question			
Overall, I enjoyed the course	5.00	4.68	−0.32 (− 0.53--0.08)
Overall, the course improved my knowledge of computers and eHealth	4.94	4.95	0.01 (−0.15–0.16)
Overall, the course gave me the experience/skills I wanted or needed	4.89	4.95	0.06 (−0.12–0.24)
Overall, the course met my learning needs	4.83	4.74	−0.09 (− 0.36–0.19)
Overall, the learning experience was better than expected	4.89	4.68	−0.21 (− 0.57–0.19)
Overall, the content of the course was easy to follow	4.94	4.37	−0.57 (− 0.92--0.17)
Overall, I am satisfied with the pace of the course	4.67	4.00	−0.67 (−1.31–0.08)
Overall, I am satisfied with the way the course was delivered	4.94	4.32	−0.62 (−1.21–0.24)
Overall, the skills and knowledge acquired during the course will help me in my job	5.00	4.84	−0.16 (− 0.39–0.91)
Overall, the course is very useful for Health Surveillance Assistants (including Senior HSAs/Supervisors and Environmental Officers)	5.00	5.00	–

## Discussion

To our knowledge, this is the first study comparing blended learning with traditional, face-to-face, class-based learning for the acquisition of ICT skills among CHWs in a resource-poor setting. Our findings show that HSAs’ knowledge and attitudes improved after attending the blended learning and traditional “Introduction to ICT and eHealth” courses. Starting with the self-rated ICT knowledge scale, our analyses revealed that HSAs’ perceived knowledge had significantly improved after the course. The larger increase was in the traditional learning group, which may be explained by the fact that the blended learning group had higher mean baseline self-rated knowledge scores compared with the traditional learning group. However, the ICT knowledge scale revealed that their competence was still elementary after course completion. Although ICT knowledge scores were generally higher after the course, our analyses show no significant differences in ICT knowledge acquisition in most domains across both groups. This may indicate that the course’s practical component requires further improvement. Although all HSAs had experience with mobile phones, many were unfamiliar with basic computer hardware, such as the keyboard and mouse. Group discussions revealed that both groups felt on-site training days could be extended to allow for more practical time on the use of computers.

Most importantly, our findings show that the use of self-rated scales without some objective measure in similar contexts is problematic as it may produce misleading results. Although the scales were designed to protect against response bias by using simple, clear language and negatively phrased questions, providing a simple and exhaustive set of options, and involving the target audience in the design/testing of the survey, participants provided conflicting responses. The ICT knowledge scale was designed to test whether the responses in the self-rated ICT knowledge scale are a true reflection of participants’ knowledge of computers, tablets and smartphones. For example, participants were asked to indicate whether they agree with the statement “I know how to use a word processor (Microsoft Office Word) application” (self-rated ICT scale). Participants in both groups provided a strongly positive response after each course. However, when they were asked to select the correct answer(s) from three possible options with regards to the question “I can use Microsoft Word to” (ICT knowledge scale), only two participants in the traditional learning group and one in the blended learning group provided a completely correct response (with the majority providing a partially correct response), which explains the low mean scores in both groups.

Attitudinal gains after the course were greater in the blended learning cohort compared to the traditional learning cohort, with significant increases in five domains in the blended learning group compared with two domains in the traditional learning group. This may be due to the fact that HSAs enrolled in the blended learning course had the opportunity to use the smartphone device for a longer period compared to the traditional learning group. The blended learning approach allowed HSAs to use the smartphone devices to revise in their own time, which may have contributed to their positive attitudes toward computers, tablets and smartphones.

Overall, HSAs reported a high level of satisfaction with the courses. Additional information on participants’ satisfaction with the course was collected through group discussion held at the end of each course. The discussion included open-ended questions about the usefulness of the course, its strengths and weaknesses, and areas for improvement. Participants were generally pleased with the course content and structure. They acknowledged the growing presence of eHealth within Malawi and appreciated the opportunity to learn more about eHealth and ICT. However, they also felt that the length of the course was short and more time in the computer laboratory is needed to master the practical components of the course. There were also concerns that the ICT training would be undermined by the lack of opportunities for practical experience in their workplace. Accordingly, the course content could be altered to suit the current needs of HSAs. Problems with the content of the course were more evident in the blended learning group, where participants were required to use the knowledge acquired during the lab-based ICT skills sessions to study independently, reinforcing the need for more ICT skills training in the lab.

In line with previous studies, we found that computer knowledge and utilization among community health workers in Sub-Saharan Africa is low [15, 24, 26]. Similarly, we found that the lack of infrastructure for ICT impedes the implementation of eLearning initiatives [29, 30]. Due to resource limitations and infrastructural challenges within villages, HSAs were provided with smartphones rather than laptop computers. Because a significant portion of the course content covered computer-related topics, HSAs felt that the absence of a computer at home made it more difficult for them to grasp some of the concepts.

In addition, due to lack of electricity and network coverage in many villages, the course content was delivered offline. Inadequate energy supply is a major problem in Malawi, especially in rural areas, where the majority of the population lives. The overall electrification rate in Malawi is about 10%, with 37% of the urban population and only 2% of the rural population having access to electricity [31]. Solar energy panels are often used as an energy source to charge mobile phones and other electronic devices in places with limited electricity supply. This method was used by the HSAs in the blended learning group to charge their phones. Although HSAs did not report any problems with electricity supply, it is unclear whether this had an impact on compliance with the course material. Nevertheless, the limited electricity supply presents a barrier to the implementation and sustainability of electronically mediated programmes in Malawi.

HSAs commented on the limited support that was provided during their off-site sessions. A research assistant was available to them via telephone; however, forums and email were not possible due to the lack of Internet connectivity. Overall, the negative feedback mainly concerned the logistical components of the course (e.g. lack of additional allowance for course attendance) rather than the blended learning approach itself.

While the computer skills scale was invaluable in capturing HSAs’ computer skills competency, it would be useful for future iterations of the course to additionally capture comprehension of the theoretical eHealth concepts covered in the course. Assessing learning outcomes with an exam may also be useful for the HSAs in comprehending the material as well as for researchers understanding the strengths and weaknesses of blended learning versus traditional learning.

### Limitations

Our study was limited to 20 participants per group due to the size of the Health Information Systems computer laboratory at Mzuzu University. Therefore, the small sample size of our study may have limited the generalizability of our results. In addition, HSAs in the blended learning group had a higher baseline self-rated knowledge score compared to their colleagues in the traditional learning group. The fact that the traditional learning course was administered several months before the blended learning course gives rise to suspicion that the course content may have been passed on to HSAs from the blended learning group prior to the course. HSAs in the traditional learning group were instructed to not share information about the course with their colleagues to protect against contamination; however, it is unclear whether they complied. Moreover, although the face validity of the questionnaires was tested prior to the courses, the use of a neutral (“neither agree nor disagree”) option in the self-rated ICT knowledge and attitudes scales may have undermined the integrity of those scales as respondents of the pre-questionnaire may have chosen this option simply because they did not understand or like the question as posed. Also, although respondents were instructed to select one or more options in the post-ICT knowledge scale, most (16/19 in the traditional learning group and 13/20 in the blended learning group) selected only one option, which has led to low mean scores in both groups (as their answers were considered incorrect). Finally, although the courses required the same level of commitment from both groups (31 teaching and learning hours in total), the blended learning approach enabled HSAs to spread this effort over a longer period minimising the disruption to field activities. However, we did not ask HSAs to keep a record (e.g. diary) of their activities during the course to enable accurate analysis and comparison of the differences in time commitments between the two groups.

Further research is needed to understand whether the time difference in the receipt of sessions between the two approaches had an impact on knowledge or skills, as well as to examine compliance with the mLearning material and schedule. Given that learning in the blended learning group spanned over a longer period of time, participants’ self-confidence in their knowledge or retention of information may have been affected. Additionally, while assessing the acquisition of knowledge and skills immediately after the end of a training course is important, it is even more important to assess whether such knowledge and skills are being put into practice once trainees get back to work and address any barriers and challenges that might prevent them from doing so. In addition, future studies may assess CHWs’ skills in using ICT using a performance-based assessment approach. While incorporating a direct knowledge testing scale was useful, a direct assessment at the computer lab would have been more beneficial.

Future studies may also examine the barriers and facilitators of scaling up blended learning courses for community workers in developing countries. As eHealth programmes gain popularity in countries like Malawi [32], there is capacity for training courses and eHealth programmes to leverage synergies to empower this cadre of healthcare workers. Community workers, such as HSAs, perform a wide range of duties, ranging from water sanitation assessments to disease management and surveillance [33]. Providing them with the right tools and training to perform their duties can improve the delivery of those services. However, given the shortage of health professionals in remote areas, this needs to be done with minimal disruption to their work and communities. Given that blended learning courses have the potential to meet the training needs of CHWs with less disruption compared to traditional learning, it is important to understand the factors that hinder or facilitate the uptake of such programmes in resource-poor settings.

## Conclusions

This study shows that there is no difference between blended and traditional learning in the acquisition of actual ICT knowledge by CHWs in rural areas in low and middle income settings. Given that blending learning has been found to be more cost-effective for training CHWs in Sub-Saharan Africa compared with traditional learning approaches [22], we conclude that, with adequate on-site training and support, blended learning can potentially present an advantageous alternative to traditional learning for training CHWs in remote and resource-poor settings.

## Additional files

Additional file 1: Baseline questionnaire. This pre-course questionnaire was used to collect socio-demographic data, information on HSAs’ experience with computers, tablets and/or smartphones, as well as data on HSAs’ self-rated and actual ICT knowledge and attitudes towards computers, tablets and smartphones. (DOCX 69 kb)

Additional file 2: Follow-up questionnaire. This post-course questionnaire was used to collect data on HSAs’ self-rated and actual ICT knowledge and attitudes towards computers, tablets and smartphones, as well as satisfaction with the course. (DOCX 54 kb)

## Abbreviations

CHWs: Community healthcare workers
DHO: District health officer
EHR: Electronic health record
HSAs: Health surveillance assistants
iCCM: integrated Community Case Management
ICCs: Intra-class correlation coefficients
ICT: Information and communication technologies
MDGs: Millennium Development Goals
NGOs: Non-governmental organizations
SMS: Short message service
