# Supplementary material for: Training community healthcare workers on the use of information and communication technologies: a randomised controlled trial of traditional versus blended learning in Malawi, Africa
Source: BMC Med Educ. 2018 Apr 2;18:61. doi: 10.1186/s12909-018-1175-5 (PMC5879741; doi:10.1186/s12909-018-1175-5)
Supplement: Supplementary file 1 — Baseline questionnaire. This pre-course questionnaire was used to collect socio-demographic data, information on HSAs’ experience with computers, tablets and/or smartphones, as well as data on HSAs’ self-rated and actual ICT knowledge and attitudes towards computers, tablets and smartphones. (DOCX 69 kb) [file 12909_2018_1175_MOESM1_ESM.docx]

***The Supporting LIFE Computer Utilisation, Knowledge and Attitudes Questionnaire***

# A. General information

Dear Sir/Madam,

The purpose of this questionnaire is to collect information on your existing experience, knowledge and attitudes towards computers (i.e. desktops or laptops) and other information technologies, such as tablets and smartphones. This information will be used to assess the efficacy of the Supporting LIFE ‘Introduction to Computer Skills and eHealth’ course and inform its future improvement. You are invited to fill out this questionnaire as you are a health surveillance assistant (HSA) and have agreed to participate in this course. The information you may provide will be used to inform the overall Supporting LIFE project evaluation.

In order to effectively achieve the aims of this programme, you are kindly requested to give a genuine response to each question. All information you may provide will be treated in the strictest confidence. There is no need to put your name on the questionnaire. It is entirely up to you to decide whether you answer any or all of the questions in the questionnaire. However, your response to these questions will help us to improve the quality of the training provided to you and your colleagues. If you decide to take part, please fill in the questionnaire and return it to the course tutor. If you have any queries about this questionnaire and the study, please ask the course tutor who will assist and provide you with any additional information you require.

Completion and return of this questionnaire means that you have consented for the research team to use your anonymised answers in reports or publications arising from the study.

If you are agreeable to participate in the study please visit the next page. Thank you for participation in this study.

**B. Socio-demographic data**

*(Please select* ***one answer*** *and where appropriate* ***specify a value*** *for each category below)*

| No | QUESTIONS | ANSWERS |
| --- | --- | --- |
| 1 | Age in years | 1. Please specify:___________ 2. Prefer not to say |
| 2 | Sex | 1. Male 2. Female 3. Prefer not to say |
| 3 | Educational status | 1. Primary education 2. Secondary education 3. Diploma or certificate 4. BSc or other University degree 5. Postgraduate (Master’s) degree 6. PhD 7. Other, specify:_________ 8. Prefer not to say |
| 4 | Marital status | 1. Single 2. Married 3. Divorced 4. Widowed 5. Separated 6. Prefer not to say |
| 5 | Religion | 1. Christian - specify denomination____ 2. Muslim 3. Other, specify:_________ 4. Prefer not to say |
| 6 | Monthly income in Malawian Kwacha | 1. Please specify:___________ 2. Prefer not to say |
| 7 | Village where the clinic is based | 1. Please specify:_________ 2. Prefer not to say |
| 8 | Work position | 1. Health surveillance assistant 2. Senior health surveillance assistant 3. Environmental officer 4. Other, specify:___________ 5. Prefer not to say |
| 9 | Experience in service years | 1. Please specify:___________ 2. Prefer not to say |
| 10 | How satisfied are you with your job? | 1. Very satisfied 2. Satisfied 3. Neither satisfied nor dissatisfied 4. Dissatisfied 5. Very dissatisfied 6. Prefer not to say |

**C. ICT experience**

*(Please select* ***one answer*** *for each of the questions below. For questions 12, 14, 17, 20 select* ***all that apply****)*

| No | QUESTIONS | ANSWERS |
| --- | --- | --- |
| 11 | Do you own or have access to a computer? | 1. Yes 2. No |
| 12 | If yes, where?    **Select all that apply**  **If no, go to question 15** | 1. At home 2. At office 3. At a friend 4. At a public library or centre 5. At a private centre or internet cafe 6. Other, specify:_______________ |
| 13 | How often do you use a computer? | 1. Every day 2. At least once a week 3. Once in a month 4. Rarely (less than once in a month) |
| 14 | I use my computer to:  **Select all that apply** | 1. Browse the web 2. Download and save content (e.g. pdf journal article) 3. Access or send emails 4. Access, create or edit a document 5. Access, create or edit a spreadsheet 6. Access, create or edit a presentation 7. All of the above 8. Other, specify: _______________ |
| 15 | Have you ever had computer training? | 1. Yes 2. No |
| 16 | If yes, what type of training did you have?  **If no, go to question 18** | 1. Short term on job training from office 2. Short term training at training centre 3. Formal training at university or college 4. Personal effort/informal training 5. Other, specify:_______________ |
| 17 | What was the training about?  **Select all that apply** | 1. Introduction to computers 2. Microsoft Office Word 3. Microsoft Office Excel 4. Microsoft Office PowerPoint 5. Microsoft Office Access 6. Internet and email 7. All of the above 8. Other, specify:_______________ |
| 18 | Do you own or have access to a tablet or smartphone device? | 1. Yes 2. No |
| 19 | If yes, how often do you use a tablet or smartphone?  **If no, go to question 21** | 1. Every day 2. At least once a week 3. Once in a month 4. Rarely (less than once in a month) |
| 20 | I use my tablet or smartphone to:  Select all that apply | 1. Browse the web 2. Download and save content (e.g. pdf journal article) 3. Access or send emails 4. Access, create or edit a document 5. Access, create or edit a spreadsheet 6. Access, create or edit a presentation 7. All of the above 8. Other, specify: _______________ |

**D. ICT skills (A)**

*(Please select* ***one answer*** *to indicate how much you agree or disagree with each of the following statements, where “strongly agree” equals to “I know very well” and “strongly disagree” “I don’t know at all”)*

| No | QUESTIONS | ANSWERS |
| --- | --- | --- |
| 21 | I know how to start and close down a computer. | 1. Strongly agree 2. Agree 3. Neither agree nor disagree 4. Disagree 5. Strongly disagree |
| 22 | I know how to distinguish between basic computer components, such as software applications, hardware and operating systems. | 1. Strongly agree 2. Agree 3. Neither agree nor disagree 4. Disagree 5. Strongly disagree |
| 23 | I know how to navigate the Internet with a browser. | 1. Strongly agree 2. Agree 3. Neither agree nor disagree 4. Disagree 5. Strongly disagree |
| 24 | I know how to download and save content (e.g. pdf files). | 1. Strongly agree 2. Agree 3. Neither agree nor disagree 4. Disagree 5. Strongly disagree |
| 25 | I know how to use an email management tool (Microsoft Outlook). | 1. Strongly agree 2. Agree 3. Neither agree nor disagree 4. Disagree 5. Strongly disagree |
| 26 | I know how to use a word processor (Microsoft Office Word) application. | 1. Strongly agree 2. Agree 3. Neither agree nor disagree 4. Disagree 5. Strongly disagree |
| 27 | I know how to use spreadsheets (Microsoft Office Excel). | 1. Strongly agree 2. Agree 3. Neither agree nor disagree 4. Disagree 5. Strongly disagree |
| 28 | I know how to use a visual and graphical application (Microsoft Office PowerPoint). | 1. Strongly agree 2. Agree 3. Neither agree nor disagree 4. Disagree 5. Strongly disagree |
| 29 | I know how to switch on, navigate and switch off a tablet or smartphone. | 1. Strongly agree 2. Agree 3. Neither agree nor disagree 4. Disagree 5. Strongly disagree |
| 30 | I know how to navigate a mobile app. | 1. Strongly agree 2. Agree 3. Neither agree nor disagree 4. Disagree 5. Strongly disagree |

**E. ICT skills (B)**

*(Please select* ***all that apply*** *from the following statements if you currently use or if you have ever used a computer. Select “Don’t know”* ***if you have never used*** *a computer)*

| No | QUESTIONS | ANSWERS |
| --- | --- | --- |
| 31 | The ‘start menu’ allows you to: | 1. Start programmes 2. Turn off the computer 3. Search for files, folders and programmes 4. Don’t know |
| 32 | Which of the following is an example of an operating system? | 1. Microsoft Office 2. Windows XP 3. Central processing unit (CPU) 4. Don’t know |
| 33 | Which of the following is an example of an Internet browser? | 1. Google Chrome 2. Mozilla Firefox 3. Youtube 4. Don’t know |
| 34 | I can download and save a journal article as: | 1. Pdf file 2. MP3 audio file 3. Flash Video (FLV) file 4. Don’t know |
| 35 | I can use Microsoft Outlook or Yahoo Mail or Gmail or Hotmail to: | 1. Create, forward or print an email 2. Create a video file 3. Create a calendar meeting or appointment 4. Don’t know |
| 36 | I can use Microsoft Word to: | 1. Create, edit and print a document 2. Create a calendar meeting or appointment 3. Insert pictures into a document 4. Don’t know |
| 37 | I can use Microsoft Excel to: | 1. Enter, filter, sort and chart data 2. Create and edit a formula 3. Create slides for a presentation 4. Don’t know |
| 38 | I can use Microsoft PowerPoint to: | 1. Create audio files 2. Create a presentation, edit slides and view a slideshow 3. To insert videos and animations into slides 4. Don’t know |
| 39 | I can use a tablet or smartphone to: | 1. Access and browse the web 2. Send and receive emails 3. Download apps 4. Don’t know |
| 40 | I can use a health mobile app to: | 1. Take clinical measurements (e.g. breathing rate, vital signs) 2. Enter socio-demographic data 3. Answer clinical questions (e.g. presence and duration of symptoms) 4. Don’t know |

**F. ICT attitudes**

*(Please select* ***one answer*** *to indicate how much you agree with each of the following statements)*

| No | QUESTIONS | ANSWERS |
| --- | --- | --- |
| 41 | I believe computers, tablets and smartphones are useful in my everyday (non-work related) life. | 1. Strongly agree 2. Agree 3. Neither agree nor disagree 4. Disagree 5. Strongly disagree 6. Don’t know |
| 42 | I believe computers and mobile apps can assist me in my work at the health centre. | 1. Strongly agree 2. Agree 3. Neither agree nor disagree 4. Disagree 5. Strongly disagree 6. Don’t know |
| 43 | I believe I would be able to use a computer or mobile app to provide patient care. | 1. Strongly agree 2. Agree 3. Neither agree nor disagree 4. Disagree 5. Strongly disagree 6. Don’t know |
| 44 | I believe I would be able to learn how to use a computer or mobile app. | 1. Strongly agree 2. Agree 3. Neither agree nor disagree 4. Disagree 5. Strongly disagree 6. Don’t know |
| 45 | I believe computers and mobile apps can support my decision making during healthcare provision. | 1. Strongly agree 2. Agree 3. Neither agree nor disagree 4. Disagree 5. Strongly disagree 6. Don’t know |
| 46 | I think using computers and mobile apps would increase my workload. | 1. Strongly agree 2. Somewhat agree 3. Neither agree nor disagree 4. Somewhat disagree 5. Strongly disagree 6. Don’t know |
| 47 | I think the use of computers and mobile apps would improve quality of care. | 1. Strongly agree 2. Agree 3. Neither agree nor disagree 4. Disagree 5. Strongly disagree 6. Don’t know |
| 48 | I do not have time to learn how to use computers and mobile apps. | 1. Strongly agree 2. Agree 3. Neither agree nor disagree 4. Disagree 5. Strongly disagree 6. Don’t know |
| 49 | I do not have time to use computers and mobile apps. | 1. Strongly agree 2. Agree 3. Neither agree nor disagree 4. Disagree 5. Strongly disagree 6. Don’t know |
| 50 | Overall, I believe using computers and mobile apps in patient care is a good idea. | 1. Strongly agree 2. Agree 3. Neither agree nor disagree 4. Disagree 5. Strongly disagree 6. Don’t know |

**Thank you for participating in this study!**
